# Dental Education

**Published:** 1873-11

**Authors:** G. R. Thomas


### DENTAL EDUCATION.

BY G. R. THOMAS, D. D. S.

In attempting to write upon a subject so full of interest and of so wide a scope, one is overcome with the extent of the undertaking, and in the present paper I shall only hope to make a few suggestions which may enlarge our field of vision and lead us to new thoughts for ourselves. The education of a person for any calling in life, depends almost entirely upon the person himself; aud according as he is possessed of energy, courage, perseverance, a love of his occupation, and last, but not least, true gentlemanly courtesy, so in proportion will he be likely to succeed. One may not be possessed of actual genius and yet be successful, but the other qualities I have named seem absolutely essential.

First, then, energy seems to be a requisite. How many men do we see, contented with the droppings from other men's hands, and too weak to overcome the obstacles necessary to perfect themselves for active pursuits! True energy mingled with courage are priceless endowments for a young man beginning in business, very many are the rough and stony places which these two attributes will help him to pass in safety. Many are the times with nearly every man when "thick dark ness" seems about to envelop him; but courage comes to his rescue, strengthens his energy, and he *perseveres*. The third characteristic then follows the other two. We have all seen many men select a calling in life, follow it a short time, *become discouraged*, leave it and try something different, which in time they also give up. What shipwreck they make of life! "Driven of the wind and tossed," they are at length stranded on the shores of time, and people point them out and say that was a staunch, trim ship once, but now even the old timbers are fit for nothing but the "drift-wood fire."

In starting in any business, a man should examine it thoroughly beforehand and see if he has fitness for it, if he thinks it will be congenial to his tastes, and if he can bring his energies to bear upon it. We will suppose a young man, with these qualifications, has decided upon the practice of dentistry. With these, he should be possessed of a good constitution and great powers of endurance ; for those of us who have been long in the profession can testify to the wearing effect it has upon both mind and body. Who of us has not experienced the intense fatigue consequent upon protracted operations at the chair ? And when continued day after day, it will tell upon the *strong* man, and much more surely on one delicately organized. There are few indeed, of our professional brethren, who have been able to stand against the constant drain upon the energies of the system. For this cause it is my firm belief that it is an impracticable idea for women to enter the practice of dentistry. There may be a very few exceptional cases, but, as a rule, those very characteristics which it is argued would make superior dentists of them—i.e. keen perceptive faculties, delicacy of touch and the great preponderance of nerve over physical force, would soon be the cause of complete failure of health. The constrained position the operator is obliged to assume, and continue in for hours together, would, doubtless, under certain circumstances, prove very disastrous and perhaps fatal to a female operator. There are a few women, who have perhaps sufficient nerve to engage in this profession, who are willing to live in single blessedness, but for a married woman such an effort would prove utterly futile.

The man who takes up a profession, simply with the idea that it will bring him a livelihood, who has no love for it, no enthusiasm in it, will never make much progress ; he will plod along from year to year, and do as little as he can for the money. But he who puts his heart into his handiwork, who every day seeks to improve on the day before, and looks with true pride on his achievements as they grow under his hands, will truly succeed. There are different phases presenting themselves to the practitioner with every new patient, and developments of disease which may be utterly strange, and will require great delicacy of treatment. Think you that man who has left the shoemaker's bench or the blacksmith's anvil and studied long enough to make a tolerably looking plate of teeth, is fitted to undertake the treatment of those delicate cases on which comfort, and perhaps life depend ; and yet, many do so, unshrinkingly and without a blush. The idea that the mere putting up of artificial teeth, or inserting a plastic, (*or gold filling, if you please*,) is all there is to dentistry, has been of greater injury to the profession than can be repaired probably in our day.

The beginning of study for dentistry is not how to fit a plate of teeth into a mouth, but to study the physiology and anatomy of the human system. In the medical profession, no one is deemed entitled to confidence who has not fitted himself for it by a course of study ; and he who would attempt to treat the delicate organs of vision, without proper study, would be set down by intelligent people as a mere quack, unworthy of notice : and is ophthalmology of so much greater importance than dental pathology, that any one who can extract a tooth can be dubbed Doctor, and entrusted with the care of those delicate organs on which so much of comfort depend? I trust the time is not far distant, when every dentist shall be a physician ; not that every physician should be a dentist, for it is a specialty by itself as certainly as is ophthalmology. The infinite number of nerves and their minute ramifications, the intimate connection between the organs of the mouth and stomach, render it a matter of vast importance that the dentist should be able to make as correct a diagnosis of any case under his care as would a physician. In administering anæsthetics, the best good sense and judgment are necessary in addition to theoretical knowledge, in order to prevent the serious, and often fatal results, which frequently follow in the hands of inexperienced operators.

The dentist needs to educate in himself certain qualities which will render him congenial to his patrons, and also to his professional brethren. In establishing an office he should make in the beginning, the resolve—*and keep it too*—that order, and scrupulous neatness shall reign there. His own person should be well attended and neat in every particular, his hands faultlessly clean, and this applies to every one of the fraternity, whether young or old. He should never go from one patient o another without washing his hands, and always keep a plentiful supply of clean towels and napkins for his own use and that of his patients. Instruments should always be thoroughly cleaned after *each* and *every* operation and lain away out of sight. Many seem impressed with the idea that they must have all their instruments on exhibition in order to impress those who come in with the number and variety of their appliances. This is a mistake, it is enough for a patient to know that a painful operation is to be performed, without being greeted with a full view of the instruments of torture. The office should be as near as possible like a private room, and as attractive as the means of the proprietor will permit. There are those, who yearly expend for tobacco and liquors that which, laid out in their places of business, would improve them vastly, and by divesting themselves of the unpleasantness which is always consequent upon these habits would increase the comfort, *if not the number*, of their patients. What lady of refined tastes would go a second time to an operator whose breath betokens a mouth fairly tanned by the use of tobacco? No one in their right mind would trust either a medical or dental practitioner who habituates himself to the use of liquors. If either of these habits have become too strong to be broken off entirely, they should at least not be indulged in at such times as will prevent the possession of a sweet, clean breath when approaching a patient. There are none of us who do not shrink with loathing from a bad breath, even when taken casually, and how much worse when subjected to it during a protracted operation. Keep a sweet breath, then, by all means.

What greater shock can a delicate and nervous woman or a frightened child receive, on entering an office to have a much-dreaded operation performed, than to be met by harsh words or rough treatment. There are often patients who try the good nature of the practitioner to the utmost, yet how earnest is the gratitude frequently expressed after a painful operation, in return for the forbearance which merely cost an effort of the will, and it is not that we should school ourselves to gentleness and patience, only with people of wealth and influence, whose patronage we may feel anxious to retain, but also toward the humbler ones. We all know that wealth is not always the guaranty of worth. In all cases, he who possesses true gentlemanly courtesy, and exercises it, will himself be benefited.

Far too many in our profession, as in others, allow themselves to indulge in detraction of their professional brethren. This is so common that it is proverbial, but how much worse than useless is it to attempt to injure the business reputation of a man because he is of the same occupation as ourselves! Suppose, for an instant, that you are the only one of your profession in your place; could you by any possibility do all that would be required of one, or even two men, and if you could, is it an honorable spirit to wish to deprive a fellow man of the privileges you claim for yourself? The world is large enough for us all, and if we see a brother prospering, why not rejoice with him, and seek to bring about that spirit that will lead to an interchange of ideas, not only at dental associations, but also among those who live and practice in your own society; never allow a word to pass your lips, against a cotemporary, unless you have incontestible proof that he is imposing on the community, and likely to do great harm; do not speak evil of any one because they are building up a good practice and are likely to succeed.

Let us, as members of a profession as yet in its youth, seek to increase its influence and usefulness, by endeavoring to raise the standard of excellence, and as wise master builders lay upon a good foundation the gold, silver and precious stones which shall stand the test of time, and thus encourage those who follow us to build higher and more skillfully, till our noble structure, though begun in weakness, shall be crowned by the dome of perfection and be honored by all nations.

